# Implementation: The Next Giant Hurdle to Clinical Transformation With Digital Health

**DOI:** 10.2196/16259

**Published:** 2019-11-20

**Authors:** Lorraine Buis

**Affiliations:** 1 Department of Family Medicine University of Michigan Ann Arbor, MI United States

**Keywords:** digital health, digital medicine, ehealth, mhealth, implementation, knowledge translation, publishing, open access, journalogy

## Abstract

Clinical implementation of digital health is a major hurdle to overcome in the coming years. Considering the role of the *Journal of Medical Internet Research* in the past 20 years and looking toward the journal’s future, this viewpoint acknowledges the vision of medicine and the role that digital health plays in that vision. It also highlights barriers to implementation of digital health as an obstacle to achieving that vision. In particular, this paper focuses on how digital health research must start looking toward implementation as an area of inquiry and the role that the *Journal of Medical Internet Research* and its' sister journals from JMIR Publications can play in this process.

Digital health is quickly revolutionizing the way health care is conducted. For two decades, the *Journal of Medical Internet Research* (JMIR) has been the premier outlet for publishing digital health research, and as the field has grown more sophisticated, so has the research itself. Starting with studies of health-related websites and evolving to online communities with message boards, Web-based interventions, social networking sites, mobile health interventions, and remote sensing, JMIR has helped build the evidence base, from descriptive studies to randomized controlled trials.

As we take a moment to reflect on where we have been and look to the future to see where we are going, the shared vision of health care is clear. Without a doubt, digital health is here to stay. Digital technologies supporting the collection of patient-generated health data, and algorithms and tools for interpreting and managing the copious amounts of data, will serve to put real-world data into the hands of health care providers who can act on it in real time. Digital health has the potential to connect individuals to patients, providers, and data; assist with self-management and behavior change; level the playing field to reduce health disparities and improve access to care; and surveil and disseminate information for public health, including in times of disaster. If all goes according to plan, digital health will accomplish these aims as well as improve efficiency in both clinical workflow and cost. Although this future vision of health care is anticipated and hoped for by many, and ongoing work today is demonstrating the feasibility of digital health in accomplishing these aims, the road to how we achieve this vision still unknown.

To illustrate, one question that has emerged in my own work is how to fit effective digital health interventions into existing clinical practice. As researchers in an emerging field, we tend to put technology into the hands of doctors and nurses to ask a research question about how to improve health outcomes. There are thousands of studies in the literature reporting feasibility, acceptability, and effectiveness of digital health interventions. However, we do not have the answers to how we could make effective technologies practicable in clinical settings, which is the piece needed to make our future vision of health care a reality.

With roots in the area of diffusion of innovations, popularized by Everett Rogers in the 1960s [1], the focus on dissemination and implementation research within health care contexts has been rapidly gaining momentum over the past few decades [2]. Although these concepts are not new, the increasing focus on factors that affect translation of evidence-based interventions, practices, and policies into real-world settings within health care has brought dissemination and implementation research to the forefront of our national research conversation. It is increasingly understood that it is not enough to just push a new development into the world and hope it sticks. Regarding digital health, researchers are just starting to look at implementation strategies themselves to find efficient ways to enhance implementation efforts [3-5] as well as to evaluate digital health implementations to understand why interventions perform the way they do [6]. Naturally, questions arise, such as how do we fit these technologies into existing workflow practices where providers do not have the time or training to utilize them effectively within clinical practice? Who will serve to troubleshoot problems experienced by providers and patients? Who will help patients, particularly less savvy ones, get up and running? How will providers fit the use of these technologies into already packed clinical encounters? How will health systems pay for these technologies? What are the ethical considerations that must be made before widespread dissemination can occur? Issues such as these are where the rubber meets the road and will make or break our realization of a shared vision of health care. Without answers to these questions, our vision is just a dream.

To meet the demands of our future needs related to digital health, one implementation strategy might be to have existing medical professionals such as medical assistants, nurses, physician assistants, or others shift roles to include new tasks created by the introduction of new technologies within health care. However, it is possible that if we were to fast forward 20 years, we may see an entirely new class of health care providers: tech-savvy clinical professionals who assist with patient-generated health data and remote sensing, are specially trained to monitor dashboards of patient-generated health data, and are assisted by algorithms to detect issues related to safety. These professionals, embedded in the health care systems they serve, would be uniquely poised to communicate with care teams about changes in conditions and recommend acute or routine follow-up. Much like the introduction of medical scribes within clinical care as a way to manage documentation burdens imposed by electronic health record systems [7], specially trained personnel who can help manage burdens associated with the collection of patient-generated health data may be useful.

Although it is intuitive that the development of a role such as this would be helpful, in the current publishing landscape, it is unclear what publication outlets would consider research into this type of implementation strategy to be in scope. As is often the case with this kind of multidisciplinary work, this work could have many possible homes including journals on practice management, clinical informatics, medicine, nursing, and public health; however, it does not squarely fit the mold of what one would expect in discipline-specific publications. This is exactly why JMIR (and its sister journals) has become, and will continue to be, the pre-eminent home for research on digital health. JMIR has been publishing manuscripts related to focused implementation of digital health technologies for two decades. From papers focused on lessons learned [8] to mixed methods studies looking for a deeper understanding of digital health in practice [9], studies of which delivery modality works best [10], and understanding characteristics of digital health among nonusers/nonadopters and users/adopters and reasons for continued engagement or dropout to gain a deeper understanding [11-17], JMIR journals have been a welcoming home for this work.

In the coming years, we will continue to have the need for research detailing efficacy and effectiveness; however, more research exploring optimal configuration of interventions, implementation strategies, cutting-edge evaluation methods for digital health, and more widely focused research centered on complex implementation issues is certainly on the horizon. As we look toward the next two decades and beyond, implementation issues are paramount, and through dissemination in one of the many journals within the JMIR family or a new JMIR publication yet to be developed, implementation will surely become a critical topic of interest to the readers, yielding yet another opportunity for JMIR to continue leading the way.

## Abbreviations

JMIR: Journal of Medical Internet Research
